# Minimizing Travel Burden of Gynecologic Cancer Surveillance Through a Unique Multidisciplinary Telehealth Program

**DOI:** 10.1097/og9.0000000000000129

**Published:** 2025-10-23

**Authors:** Avni Shridhar, Suzanne Viator, Tara Castellano, Holly Provost, Navya Nair, Elizabeth Neupert, Amma Agyemang, Amelia Jernigan

**Affiliations:** Section of Gynecologic Oncology, Department of Obstetrics and Gynecology, School of Medicine, Louisiana State University Health Sciences Center New Orleans, and the Department of Gynecologic Oncology, University Medical Center New Orleans LCMC Health, New Orleans, and the Section of Gynecologic Services, Ochsner University Hospital & Clinics, Lafayette, Louisiana; and the Section of Gynecological Oncology, Department of Obstetrics, Gynecology and Reproductive Sciences, Miller School of Medicine, University of Miami, Miami, Florida.

## Abstract

**OBJECTIVE:**

STEEL MAGNOLIAS (shared telehealth for multidisciplinary gynecologic cancer survivorship) is a novel gynecologic cancer surveillance care–delivery program in which rural patients see a close-by gynecologist in person with simultaneous virtual gynecologic oncology consultation. This study assesses feasibility of STEEL MAGNOLIAS by examining travel burden reduction, cancer outcomes, visit activities, guideline adherence, and patient satisfaction.

**METHODS:**

We retrospectively reviewed charts of patients with gynecologic cancer in remission under the STEEL MAGNOLIAS program in rural south Louisiana (March 2020–September 2023). Travel metrics, patient satisfaction, cancer outcomes, survival status, reasons for visit, and adherence to follow-up as per National Comprehensive Cancer Network (NCCN) guidelines were recorded. A small subset of patients prospectively completed questionnaires, such as the COST-FACIT (Comprehensive Score for Financial Toxicity-Functional Assessment of Chronic Illness Therapy) for financial toxicity and the PSQ-18 (Patient Satisfaction Questionnaire Short Form), and responded to questions about existing barriers to care. Descriptive statistics and Wilcoxon rank sum tests for continuous data were used. We assessed feasibility by assessing continued patient compliance without erosion of satisfaction.

**RESULTS:**

Sixty-three patients attended 178 STEEL MAGNOLIAS appointments, and most of the patients had a history of endometrial cancer. The majority, 82.5%, were alive with no evidence of disease. Our patients traveled a median of 16.9 miles for STEEL MAGNOLIAS, compared with 137 miles for in-person visits. We identified prevalent transportation and technology barriers to in-person and conventional virtual visits. Patients demonstrated high satisfaction with appointments. Genitourinary and cancer therapy symptoms and reviews of laboratory test results and imaging often were discussed. Laboratory tests, imaging, and referrals were ordered and completed at high rates, with 76.9% of appointments adhering to NCCN follow-up guidelines.

**CONCLUSION:**

The STEEL MAGNOLIAS program is a feasible, innovative hybrid telehealth model for rural gynecologic cancer surveillance that reduces travel burdens and ensures high guideline adherence and patient satisfaction. This scalable model has potential to improve outcomes and compliance, meeting patients where they are and transforming cancer survivorship.

## Abstract 2

A unique multidisciplinary telehealth program is feasible, provides quality gynecologic cancer care, and significantly reduces travel burden for rural patients.

After patients are treated for gynecologic cancer, surveillance visits detect recurrence of disease and optimize quality of life but may be challenging for some individuals. These visits are recommended as frequently as every 3 months for comprehensive history and physicals, diagnostics, and survivorship education.^1^ The travel required is particularly burdensome for rural patients.^2^ One-third of U.S. counties are more than 50 miles from the nearest gynecologic oncologist.^3^ Rural patients have less access to quality care, poorer outcomes, and higher mortality.^4,5^ As cancer outcomes improve, enhancing survivorship and access to quality care requires that we explore alternatives for patients far from high-volume centers.

Telehealth has emerged as a pivotal health care–delivery tool, offering cost-effective and convenient access to specialists.^6,7^ Emerging studies suggest that in oncology settings such as gynecologic cancer surveillance, critical markers of recurrence such as tumor markers, imaging results, and patient-reported symptoms can be effectively monitored through virtual consultations.^8^ Still, telehealth poses barriers to access.^9–11^ Virtual visits often fail to meet National Comprehensive Cancer Network's (NCCN) surveillance standards, especially regarding recommendations for pelvic examinations.^1^ Patients report that telehealth limits their ability to express concerns, form connections with care teams, or feel secure and private.^12^

The STEEL MAGNOLIAS program (shared telehealth for multidisciplinary gynecologic cancer survivorship) aims to address these gaps through hybrid in-person and virtual models. Patients visit their close-by gynecology clinic for a complete in-person visit with complete physical examination and simultaneously participate in a virtual visit with their gynecologic oncologist on a clinic-supplied tablet. Here, we describe the feasibility and appeal of using the STEEL MAGNOLIAS model for gynecologic cancer surveillance by describing travel burden reduction, patient satisfaction, cancer outcomes, and compliance with guideline-adherent care while maintaining satisfaction.

## METHODS

This study was largely a retrospective chart review of patients with gynecologic cancer under surveillance in the STEEL MAGNOLIAS program in rural south Louisiana from March 2020 to September 2023. The STEEL MAGNOLIAS program was created in response to the challenges faced during the initial months of the coronavirus disease 2019 (COVID-19) pandemic, when rural patients were hesitant to travel to New Orleans for care given the city's hot spot status. The program was based at a single gynecologic oncology center in New Orleans, in conjunction with a general gynecology clinic located in Lafayette, Louisiana, a smaller urban center approximately 135 miles west of New Orleans that serves as a critical access point for patients from a broad, medically underserved rural region. During STEEL MAGNOLIAS visits, rural patients in surveillance for gynecologic cancer are seen in-person by the close-by gynecologist; the local clinic provides patients with internet and a tablet to facilitate a hybrid virtual visit with the gynecologic oncologist. Occasionally, patients are given the option to conduct their appointments from home on a personal device, if feasible and if an in-person clinic visit is deemed unnecessary, with most visits conducted in the hybrid fashion.

The program followed key principles of the DIMeS (definition, identification, measurement, selection) framework, focusing on tailored implementation, effective dissemination to rural partners, and long-term sustainability of care delivery.^13^ When a rural patient was placed in surveillance for a gynecologic cancer, the gynecologic oncology team at the high-volume center would refer patients to the STEEL MAGNOLIAS program. A nurse navigator situated at the high-volume center coordinates the patients and return visits with another nurse navigator at the close-by gynecology clinic. Each participating site designated an existing clinic nurse as the patient navigator who was trained in the shared-care workflow and was responsible for coordinating appointments, facilitating communication, and supporting patient follow-up and logistics across clinical sites. For the hybrid appointments, both nurse navigators would ensure that laboratory test results and images from the close-by hospital were uploaded into the system at the high-volume center for review by the gynecologic oncologist before the appointment.

The STEEL MAGNOLIAS appointments were held every Wednesday morning. The patient would present to the close-by gynecology clinic for an in-person visit. They were seen in the standard fashion by the gynecology team, which often included a full physical examination including pelvic examination, if indicated. The gynecology team would notify the gynecologic oncologist by phone to sign on for the STEEL MAGNOLIAS appointment. Using a hospital-provided tablet and Zoom, the team would have a multidisciplinary conversation, including gynecologic oncology expertise, with regards to the interpretation of imaging and laboratory results, triage and work up of abnormal examination findings, management of symptoms related to cancer and cancer therapy, and survivorship. The gynecologic oncologist would communicate back to the close-by gynecology team with recommendations in real time for any further treatment, workup, and follow-up. Gynecology and gynecologic oncology were treated as distinct specialties and billed separately, consistent with multidisciplinary care norms; each physician documented their respective components of care, with the gynecologic oncologist billing for a telehealth encounter and the local gynecologist billing for an in-person outpatient visit.

At the very start of our program, we obtained IRB approval from the Louisiana State University Health Sciences Center New Orleans IRB under protocol No. 1231: STEEL MAGNOLIAS: Shared Telehealth for Multidisciplinary Gynecologic Cancer Survivorship in remote settings—A pilot study. This initial approval allowed us to assess barriers to care, patient satisfaction, and financial toxicity in a subset of 20 patients from Lafayette under surveillance with our gynecologic oncology team using the PSQ-18 (Patient Satisfaction Questionnaire Short Form 18) and the COST-FACIT (Comprehensive Score for Financial Toxicity-Functional Assessment of Chronic Illness Therapy) questionnaires. These are validated surveys to assess patient satisfaction and financial distress, respectively.^14,15^ The COST-FACIT measures financial distress in patients with chronic illnesses, particularly cancer. The COST-FACIT score ranges from 0 to 44, with lower scores indicating greater financial toxicity. The score is derived from patient responses to 11 items that assess financial strain such as concerns about medical costs, the effect of expenses on well-being, and the ability to afford treatments and daily necessities. Higher scores suggest lower financial distress and better financial well-being. This analysis compared COST-FACIT scores between two distinct groups: the 15 patients who participated in hybrid STEEL MAGNOLIAS appointments (seen in person by a local gynecologist with virtual input from a gynecologic oncologist) and the five patients who traveled to New Orleans for traditional in-person surveillance with the gynecologic oncology team. Wilcoxon rank sum tests were used for comparison, with statistical significance defined as *P*<.05. We defined feasibility as 75–85% compliance without erosion in satisfaction.

After a few years of conducting clinical care with this pathway, we modified our IRB approval to perform a retrospective chart review of all patients enrolled in STEEL MAGNOLIAS. Study data were collected using REDCap electronic data capture tools hosted at our institution.^16,17^ All patients who had at least one STEEL MAGNOLIAS visit were included, and all of their STEEL MAGNOLIAS visits during the study period were analyzed. Demographics, travel metrics, cancer outcomes, survival status, topics discussed at appointments, and adherence to NCCN guidelines for follow-up were abstracted from the chart. The inclusion of race and ethnicity were essential because the study focuses on describing a unique, intersectional rural population; detailed health care–delivery data are critical to understanding and addressing this population’s specific needs. We used race and ethnicity details that had been populated into patients' Epic Systems medical records. For appointments that required travel, Google Maps was used to estimate travel time between the patient's home address and the site where they were seen in average 8:00 am traffic conditions. *Adherence* was defined as adherence to NCCN guidelines for follow-up appointments and physician's orders for imaging, laboratory tests, and referrals. Descriptive statistics were used to summarize the findings.

## RESULTS

Demographics of the 63 patients with gynecologic cancer included in this study are listed in Table 1. The median age of patients at the time of their first STEEL MAGNOLIAS visit was 63.7 years (interquartile range 53.9–69.0). Most patients were Black (49.2%) or White (44.4%). Most were English-speaking (95.2%), with some Spanish (3.2%) and Vietnamese (1.6%) speakers. The median body mass index (BMI, calculated as weight in kilograms divided by height in meters squared) was 38.6 (interquartile range 33.3–46.8). Endometrial cancer (61.9%) was the most common diagnosis and most patients (57.1%) were stage I; 20.6% were advanced stage III or IV. Patients received a variety of treatments, with surgery (90.5%), radiation (34.9%), and chemotherapy (30.2%) being the most common.

**Table 1.** Demographics of Patients at the Time of Their First STEEL MAGNOLIAS (Shared Telehealth for Multidisciplinary Gynecologic Cancer Survivorship) Visit (N=63)

Characteristic	Value
Age at 1st visit (y)	63.71 [53.87–69.01]
Ethnicity	
Hispanic or Latina	2 (3.2)
Not Hispanic or Latina	61 (96.8)
Race	
Asian	1 (1.6)
Black	31 (49.2)
White	28 (44.4)
Unknown or not reported	3 (4.8)
1st language	
English	60 (95.2)
Spanish	2 (3.2)
Vietnamese	1 (1.6)
BMI (kg/m^2^)	38.60 [33.30–46.80]
Type of cancer*	
Endometrial	39 (61.9)
Ovarian, fallopian tube, or primary peritoneal	11 (17.5)
Cervical	7 (11.1)
Uterine sarcoma	5 (7.9)
Vulvar	2 (3.2)
Vaginal	1 (1.6)
Other	6 (9.5)
Highest confirmed stage	
I	36 (57.1)
II	6 (9.5)
III	12 (19.0)
IV	1 (1.6)
Unknown	8 (12.7)
Treatment received*	
Surgery	57 (90.5)
Radiation therapy	22 (34.9)
Chemotherapy	19 (30.2)
Hormonal therapy	5 (7.9)
Other targeted therapy	4 (6.3)

BMI, body mass index.

Data are median [interquartile range] or n (%).

* Patients were assigned to more than one category if applicable.

Table 2 describes cancer outcomes for the patients enrolled in STEEL MAGNOLIAS. The vast majority (87.3%) achieved remission after frontline therapy, with the rest being under surveillance without remission. Ultimately, 10.9% of patients in remission experienced recurrent disease while enrolled in the program, of whom 50.0% had ovarian or fallopian cancer (n=3) and 50.0% had cervical cancer (n=3). At the time of data collection, all patients initially enrolled were alive, with 82.5% having no evidence and 17.5% with evidence of disease. Over the period of data collection, 12.7% of those enrolled were *lost to follow-up*, which was defined as having been unable to contact the patient for more than 12 months.

**Table 2.** Summary of STEEL MAGNOLIAS (Shared Telehealth for Multidisciplinary Gynecologic Cancer Survivorship) Patient Outcomes (N=63)

Outcome	n (%)
Remission after frontline therapy	
Yes	55 (87.3)
No	8 (12.7)
Experienced recurrent disease if in remission	
Yes*	6 (10.9)
No	49 (89.1)
Current status	
Alive with no evidence of disease	52 (82.5)
Alive with disease	11 (17.5)
Dead	0 (0.0)
Lost to follow-up	
Yes	8 (12.7)
No	55 (87.3)

* Ovarian or fallopian (n=3), cervical (n=3).

Table 3 summarizes travel metrics for the patients in the STEEL MAGNOLIAS program. Most appointments were held in hybrid fashion (63.7%) physically at the close-by gynecology clinic, and the rest of them were held at the patient's home over video call (36.3%). The median (interquartile range) distance patients had to drive from their house to the close-by gynecology clinic was 16.9 miles (8.58–21.60 miles), and the median length of the drive was 26 minutes (18–35 minutes). The median distance (interquartile range) patients would have had to drive to the gynecologic oncology clinic in New Orleans would have been 137 miles (132–148 miles), and the and the median length of the drive would have been 140 minutes (130–150 minutes).

**Table 3.** Summary of Travel Metrics for STEEL MAGNOLIAS (Shared Telehealth for Multidisciplinary Gynecologic Cancer Survivorship) Appointments

Characteristic	Value
Originating site for visit*	
Video call from home	66 (36.3)
Close-by gynecologist's clinic	116 (63.7)
Distance from patient's home to close-by gynecologist's clinic (miles)^†^	16.9 [8.58–21.60]
Length of drive (min)	26 [18–35]
Distance from patient's home to gynecologic oncologist's clinic (miles)^†^	137 [132–148]
Length of drive (min)	140 [130–150]

Data are n (%) or median [interquartile range].

* Total N=63 patients, 178 appointments.

† Forty-five patients attended in-person appointments.

An initial survey group of 20 women under surveillance in the Lafayette area included 15 patients enrolled in the STEEL MAGNOLIAS hybrid visits and five patients who attended traditional in-person travel visits at the high-volume center in New Orleans. Demographics of this group included a median age of 57.3 years (SD 10.4), 49.2% Black, 44.4% White, and 4.8% American Indian; most had endometrial cancer (61.9%) or ovarian or fallopian tube cancer (17.5%), with 57.1% initially diagnosed at stage I. Surveying showed that many did not have access to reliable home internet (n=5, 25.0%), a smartphone (n=7, 35.0%), or a personal computer (n=8, 40.0%). Nine patients (45.0%) were unable to drive themselves to doctor's visits in their own vehicle. The mean COST-FACIT score for all types of appointments was 24.8±12.3; although there was a trend for lower financial toxicity for STEEL MAGNOLIAS, there was no significant difference between visit types (23.8 for STEEL MAGNOLIAS, 27.6 for travel visits, *P*=.661).

**Fig. 1.**
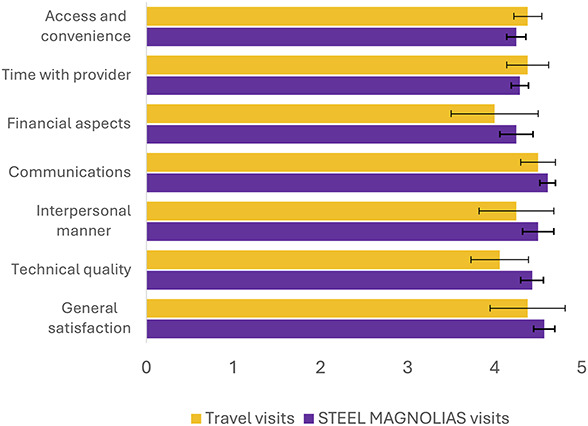
PSQ-18 (Patient Satisfaction Questionnaire Short Form) scores across each dimension of the questionnaire, presented as mean±SD using boxplots. Responses are based on a Likert scale: strongly agree (5), agree (4), uncertain (3), disagree (2), and strongly disagree (1). STEEL MAGNOLIAS visits (*purple*) refer to encounters in which patients were seen in person by a local general gynecologist with concurrent virtual consultation by a gynecologic oncologist through tablet (n=15). Travel visits (*yellow*) refer to traditional in-person surveillance appointments with a gynecologic oncologist at the high-volume cancer center in New Orleans (n=5).

The group that attended a STEEL MAGNOLIAS visit reported high levels of patient satisfaction, overall, as assessed by the PSQ-18 for one visit, similar to the satisfaction levels for those who attended in-person gynecologic oncology appointments at the high-volume center. Seven dimensions of satisfaction were assessed and are visualized in Figure 1. On average, for STEEL MAGNOLIAS appointments, general satisfaction was scored as 4.6±0.1 out of 5; technical quality as 4.4±0.1; interpersonal manner as 4.5±0.2; communication as 4.6±0.1; financial aspects as 4.3±0.2; time with physician as 4.3±0.1; and access and convenience as 4.3±0.1. For travel visits, the scores were 4.4±0.4 for general satisfaction; 4.1±0.3 for technical quality; 4.3±0.4 for interpersonal manner; 4.5±0.2 for communication; 4.0±0.2 for financial aspects; 4.4±0.2 for time with physician; and 4.4±0.2 for access and convenience.

*Shridhar. Reducing Travel for Gynecologic Cancer Care Using Telehealth. O&G Open 2025*.

Table 4 summarizes what was covered at the 178 STEEL MAGNOLIAS appointments included in this study. In terms of items addressed at the visits, preventative care was discussed at 69.6% of the appointments, and healthy lifestyle changes were discussed at 54.7% of the appointments. Patients were able to address symptoms secondary to cancer therapy at 36.5% of the appointments, genitourinary symptoms and concerns at 34.8% appointments, and findings that were concerning for disease recurrence or progression at 12.7%. In addition, new imaging studies were available and reviewed at 41.4% of appointments, and laboratory results were newly available and reviewed at 37.0% of appointments. Furthermore, the gynecologic oncologist placed orders for imaging studies at 12.1% of appointments, of which 81.8% were completed by the patient. Lab tests were ordered at 7.7% of the appointments, of which 64.3% were completed by the patient. The median time for follow-up was 6 months (interquartile range 3–6 months); among follow-up visits with sufficient time to assess adherence, 76.3% were completed as scheduled.

**Table 4.** Summary of STEEL MAGNOLIAS (Shared Telehealth for Multidisciplinary Gynecologic Cancer Survivorship) Appointment Accomplishments (N=178 Appointments)

Characteristic	Value
Issues addressed at visit other than survivorship*	
Preventative care	126 (69.6)
Healthy lifestyle changes	99 (54.7)
Imaging reviewed or interpreted	75 (41.4)
Laboratory test results reviewed or interpreted	67 (37.0)
Symptoms secondary to cancer therapy	66 (36.5)
Genitourinary symptoms or concerns	63 (34.8)
Findings concerning for disease recurrence or progression	23 (12.7)
Genetics	20 (11.0)
Decision made for a procedure	10 (5.5)
Hot flushes	6 (3.3)
End-of-life counseling	1 (0.6)
Code status	0 (0.0)
Imaging tests ordered	22 (12.1)
Completed by patient	18 (81.8)
Laboratory tests ordered	14 (7.7)
Completed by patient	9 (64.3)
Referral ordered	8 (4.7)
Genetic counseling	2 (25.0)
Interventional radiology	2 (25.0)
Radiation oncology	2 (12.5)
Gastroenterology	1 (12.5)
Medical oncology	1 (12.5)
Referral completed by patient	7 (87.5)
Time for follow-up (mo)	6 [3–6]
Adherence with next appointment by patients who have sufficient time to follow-up	
Yes	103 (76.3)
No	32 (23.7)

Data are n (%) or median [interquartile range].

* Appointments were assigned to more than one category if applicable.

## DISCUSSION

We describe our experience with the STEEL MAGNOLIAS hybrid telehealth model for gynecologic cancer surveillance, assessing the reduction in travel burden and describing cancer outcomes, patient satisfaction and compliance with guideline-adherent care in Louisiana. Demographics and cancer type and stage were consistent with expectations. We service a diverse catchment with the most common diagnosis being early-stage endometrial cancer. After treatment, 87.3% of patients achieved remission, 10.9% of whom had recurrence. A small portion (12.7%) were lost to follow-up. The model reduced travel burden, cutting median travel time by 114 minutes, and addressed barriers to virtual care such as limited access to devices and internet, which is prevalent in this population. Patient satisfaction and financial toxicity were comparable between STEEL MAGNOLIAS patients and those participating in surveillance visits that required travel to the gynecologic oncologist at the high-volume center. Over the course of 178 appointments, we addressed preventative care, symptom management, and recurrence concerns. Imaging and laboratory assessments were ordered and completed at high rates. Follow-up visits were completed as scheduled in more than 75% of cases, indicating the feasibility and sustainability of this care model. The involvement of a local gynecologist appeared to add value beyond the physical examination and included assistance with care coordination, reinforcing follow-up plans, and offering in-person support to patients. The long-standing implementation of our program, as well as the successful completion of NCCN guideline-recommended surveillance tasks and maintenance of patient satisfaction, highlights the feasibility of the STEEL MAGNOLIAS in clinical practice.

Telehealth can attenuate disparities in access and improve convenience, yet barriers remain.^9–11^ In gynecologic cancer survivorship, the inability to perform pelvic examination and the potential to increase patient anxiety are key limitations.^12,18^ The STEEL MAGNOLIAS program combines in-person examinations with remote specialist input, enhancing rigor, accessibility, and adherence to follow-up as per NCCN guidelines. Notably, previous studies highlight limited data on outcomes of surgeon-conducted telehealth follow-ups for patients with gynecologic cancer^19^ and a lack of detailed documentation on telehealth modalities or health items addressed at visits.^20–22^ This study contributes to the growing body of telehealth research by providing granular outcome data, offering insights into health care delivery at these visits, patient adherence, and satisfaction. Telehealth research often underrepresents marginalized groups, and rural residence is a complex risk factor that warrants local-level analysis.^23^ This work provides insight into our unique rural Southeastern cancer surveillance patients and underscores hybrid models' potential to bridge access disparities effectively.

As telehealth and telehealth policies continue to grow, so does the potential of virtual care to expand access to gynecologic oncology services, supporting surgical consultations, chemotherapy monitoring, and clinical trial participation, and ultimately laying the groundwork for innovative models such as STEEL MAGNOLIAS.^24^ Our program has promising implications for reshaping gynecologic cancer surveillance. By reducing travel burden while ensuring adherence to care protocols, the hybrid model could serve as a scalable, patient-centered framework. Clinicians and policymakers can embrace this model to provide guideline-adherent care closer to patients, improving outcomes, reducing travel burden, and optimizing adherence with guidelines. Equipping local clinics with resources for specialist collaboration may bridge expertise gaps in underserved regions, supporting local doctors in providing up to date care.

Building on this study's success, future steps include researching long-term patient outcomes and broader applicability across cancer types and specialties. Further investigation is needed to understand how factors such as socioeconomic status and digital literacy influence adoption and satisfaction. The program has been built on to increase access to clinical trials for rural patients. This model could also expedite time-sensitive consultations from gynecology or medical oncology offices to gynecologic oncologists in the earliest phases of care, hastening work up and treatment times. It should be noted that most patients were early-stage endometrial cancer survivors. Although national patterns often involve referral to local gynecologists for surveillance, this is not consistently the case in our region, where many patients continue follow-up with gynecologic oncology. The STEEL MAGNOLIAS model was designed to promote a more structured, collaborative pathway that could help support transitions to community-based care when appropriate. Collaborating with intermediaries could increase local access points, ensuring more patients benefit from high-quality surveillance close to home. Advocating for policy changes to boost funding and technology support for rural health centers could enable wider implementation.

A key strength of this study is its evaluation of a pragmatic, real-world care model that addresses persistent challenges in rural cancer survivorship. The program's focus on underserved rural populations and racial and ethnic minorities enhances generalizability and relevance. Its multidisciplinary structure fosters coordinated care across settings while leveraging existing resources to minimize cost and infrastructure demands. Adherence to NCCN follow-up guidelines and a longitudinal design further support the feasibility and acceptability of the approach over time. In addition, the use of validated tools to assess patient satisfaction and financial toxicity strengthens the rigor of our findings.

Limitations must be considered, however. The retrospective design, reliance on electronic medical record documentation, small sample size, and geographical specificity may limit generalizability. The study was underpowered to draw definitive conclusions from the prospective patient-reported questionnaires, particularly given the small and uneven group sizes due to limited travel visits during the early COVID-19 pandemic; these findings should, therefore, be interpreted as exploratory. Approximately 36% of visits were conducted by telehealth from patients' homes and typically involved only the gynecologic oncologist. Although these visits differed from the in-clinic shared-care model, they remained part of the STEEL MAGNOLIAS program and reflect real-world adaptations. They were, therefore, included in our analysis and are acknowledged here as a potential limitation. The program primarily serves English-speaking patients, highlighting a need for inclusive language resources. Although telehealth may pose barriers related to race, socioeconomic status, or age, our study did not include subgroup analyses, which represents a potential area for future investigation. Additionally, the study does not fully evaluate financial implications on patients or health care systems, preventing conclusions about cost-effectiveness.

STEEL MAGNOLIAS is a feasible, innovative approach to addressing the needs of rural gynecologic cancer survivors, combining telehealth with essential in-person care. The hybrid model reduces travel burden, retains high patient satisfaction, and maintains rigorous surveillance standards, paving the way for more accessible survivorship care. Cancer care delivery must be streamlined to improve quality of life. Everyone deserves access to subspecialty expertise and timely counseling, yet many face barriers to receiving these critical resources. As health care embraces digital solutions, models such as STEEL MAGNOLIAS can redefine best practices, ensuring high-quality, patient-centered cancer care reaches even the most remote communities.
